# Iron‐dependent lysosomal LDL oxidation induces the expression of scavenger receptor A in human THP‐1 monocytes

**DOI:** 10.1002/2211-5463.70048

**Published:** 2025-05-11

**Authors:** Martina Čierna, Richard Buchal, Martin Leníček, Amit Shachak, Jan Pláteník

**Affiliations:** ^1^ Institute of Medical Biochemistry and Laboratory Diagnostics, First Faculty of Medicine Charles University Praha 2 Czech Republic; ^2^ Present address: Bar‐Ilan University Ramat Gan Israel

**Keywords:** atherosclerosis, ferritinophagy, lipid peroxidation, lysosome, redox‐active iron

## Abstract

Atherosclerosis leading to cardiovascular diseases remains a dominant medical problem. In the early stages of this disease, the interaction between circulating monocytes and the endothelium is crucial. Monocytes and macrophages express scavenger receptor A (SR‐A), which mediates cell adhesion and subsequently uptake of oxidized low‐density lipoproteins (LDL). High iron stores in monocytes or macrophages are known to predispose individuals to atherosclerosis, however the reasons remain poorly understood. We hypothesized that a combination of iron and LDL may induce proatherogenic changes in circulating monocytes. Here, we treated a human monocytic cell line THP‐1 with isolated LDL and/or iron. A limited uptake of native LDL, but not iron or oxidized LDL, markedly induced expression of SR‐A in these cells. Both SR‐AI and SR‐AII isoforms were upregulated. The increased SR‐A was also seen at the protein level, and LDL treatment increased cellular adhesion. The induction of SR‐A by LDL was inhibited by the lysosomotropic thiol WR‐1065 and by the chain‐breaking lipophilic antioxidant butylated hydroxytoluene (BHT). The fluorescent probe BODIPY C11 exhibited increased lipid peroxidation inside lysosomes after LDL administration. The induction of SR‐A by LDL was blocked by two silencing RNAs directed against the nuclear coactivator receptor NCOA4, the cargo receptor necessary for the autophagy of ferritin. These results may point to a new pathogenetic mechanism of early‐stage atherosclerosis, in which high iron stores in circulating monocytes, through increased lysosomal lipid peroxidation, may lead to an upregulated expression of SR‐A, which makes the cells more adhesive and hence more atherogenic.

## Graphical abstract

In human monocytic cells THP‐1, a limited uptake of native—not oxidized—LDL/VLDL induced expression of scavenger receptor A and cellular adhesion. Induction was inhibited by lysosomotropic (WR‐1065) and lipophilic (BHT) antioxidants and by siRNAs against ferritinophagy. A lipophilic fluorescent probe showed an increased lipoperoxidation within cells. It indicates a role of iron‐mediated lysosomal oxidation of LDL in early‐stage atherosclerosis.

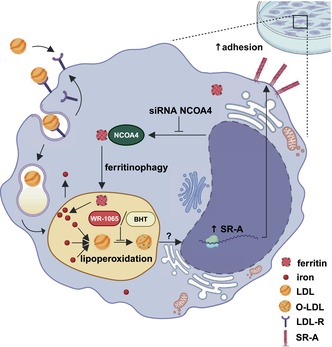

**Abbreviations**
BAF: bafilomycin
BHT: butylated hydroxytoluene
CD36: scavenger receptor B
DFO: deferoxamine
DiI: dioctadecyl‐3,3,3′,3′‐tetramethylindocarbocyanine perchlorate
FAC: ferric ammonium citrate
FBS: fetal bovine serum
GAPDH: glyceraldehyde 3‐phosphate dehydrogenase
GC: gas chromatography
HRP: horseradish peroxidase
IMDM: Iscove's modified Dulbecco's medium
IMDM‐PR−: Iscove's modified Dulbecco's medium without phenol red
LDH: lactate dehydrogenase
LDL: low‐density lipoprotein
MS: mass spectrometry
NCOA4: nuclear receptor coactivator 4
O‐LDL: oxidized low‐density lipoprotein
PBS: phosphate‐buffered saline
PMA: phorbol 12‐myristate 13‐acetate (phorbol ester)
PVDF: polyvinylidene difluoride
qPCR: quantitative polymerase chain reaction
SDS: sodium dodecyl sulfate
SIH: salicylaldehyde isonicotinoyl hydrazine
siRNA: small interfering RNA, silencing RNA
SR‐AI: macrophage scavenger receptor A, transcript variant I
SR‐AII: macrophage scavenger receptor A, transcript variant II
TBA: 2‐thiobarbituric acid
TBARS: thiobarbituric acid‐reactive substances
TBP: TATA box binding protein
TBS: tris‐buffered saline
TBST: tris‐buffered saline with Tween 20
Tf: holotransferrin
TMP: 1,1,3,3‐tetramethoxypropane
VLDL: very low‐density lipoprotein
WR‐1065: 2‐[(3‐aminopropyl)amino]ethanethiol dihydrochloride

Cardiovascular diseases are the leading cause of morbidity and mortality worldwide [1]. The primary cause of a heart attack or stroke is usually atherosclerosis, a chronic disease affecting the vessel wall of the medium and large arteries. An atheroma can develop silently for decades due to chronic inflammation, lipid oxidation, and deposition [2]. Eventually, the lesion ruptures and leads to thrombosis, obliterating the artery.

Atherogenesis starts with endothelial dysfunction and the oxidation of low‐density lipoprotein (LDL) particles as they pass from the bloodstream to tissues through the blood vessel wall. Circulating monocytes are recruited to the vessel wall and differentiate into macrophages, which act as innate immune cells and trigger or propagate local inflammation [2]. They also express scavenger receptors that are able to bind and internalize the oxidized LDL. Unlike the regular uptake of native LDL, the uptake of oxidized LDL is not subject to feedback inhibition [3, 4]. Hence, macrophages become filled with droplets of cholesteryl esters and turn into ‘foam cells’, a hallmark of atherosclerosis. At a later stage, the lipid‐laden cells die, and the lipids are also deposited extracellularly.

The scavenger receptors are a diverse group of 8 classes of structurally unrelated proteins that can bind modified LDL or other polyanionic ligands, such as bacterial lipopolysaccharides. They are employed in the removal of unwanted cells or modified proteins and in the innate immune response [5, 6]. Macrophage scavenger receptor A (SR‐A, also known as MSR), together with scavenger receptor B (CD36), remains most strongly associated with atherogenesis [7].

Epidemiological research [1] has established the classical risk factors for atherosclerosis and coronary disease, such as age, male sex, family history of coronary disease, hypertension, tobacco smoking, dyslipidemia, and diabetes mellitus. In addition, elevated body iron stores have emerged as another risk factor that significantly contributes to atherogenesis [8, 9].

Iron is obviously an essential biogenic element that is necessary for oxygen transport or storage, electron transfer, and DNA synthesis [10]. However, the key ability of iron to catalyze single electron transfers also creates potential for the generation of highly toxic reactive oxygen species unless the metal is properly shielded [2]. Atherosclerotic lesions accumulate iron, and there is solid experimental evidence that this iron contributes to atherosclerosis progression in numerous ways [11]. Iron can promote endothelial dysfunction, catalyze lipid oxidation, aggravate local chronic inflammation, induce ferroptotic cell death, and destabilize lesions [12, 13].

Macrophages are remarkably versatile cells that play a central role in the turnover of iron. They recycle iron from red blood cells and, in response to the liver peptide hepcidin, regulate its availability [14]. In fact, the body's iron is proatherogenic only if it is stored within macrophages; in the inherited iron overload condition hereditary hemochromatosis, excess iron is found in inner organs, whereas macrophages are iron‐depleted, and atherosclerosis is not accelerated [11, 15].

The actual roles of macrophages in atherosclerotic lesions are confounded by their ability to differentiate into several different phenotypes, which is known as macrophage polarization. Classical M1 macrophages are polarized after exposure to molecules such as bacterial lipopolysaccharides; these cells produce proinflammatory cytokines, easily accumulate cholesterol, and are prone to iron retention with low export capacity. In contrast, alternatively activated ‘wound‐healing’ M2 macrophages secrete anti‐inflammatory cytokines, support tissue remodeling, are less prone to accumulate cholesterol, and have a high capacity for iron uptake and export with low iron retention [11, 16].

The scavenger receptors may also come into play at the beginning of macrophage involvement in atherosclerosis—the recruitment of circulating monocytes to the blood vessel wall. SR‐A is also expressed by monocytes, albeit at minimal amounts, and mediates not only the uptake of oxidized LDL but also cellular adhesion [7]. Patients with acute coronary syndrome exhibit several times greater expression of both SR‐A and CD36 in their circulating monocytes [17]. Interestingly, SR‐A expression can be affected by the cellular iron status. In the human monocytic cell line THP‐1, it was reported that loading the cells with iron increased the cellular oxidation of added LDL, the cellular uptake of oxidized LDL, and the expression of SR‐A [18]. Can elevated intracellular iron, by increasing SR‐A expression, increase the degree to which circulating monocytes adhere to the endothelium and hence increase their atherogenic potential?

However, in our own yet unpublished experiments with THP‐1 cells, iron loading completely failed to induce the expression of SR‐A. One possible explanation for this discrepancy may be the dependence of the effects of iron on the presence of intracellular oxidized lipids. The synergy between iron and oxidized lipids in SR‐A induction has not yet been explicitly described, but it is supported by indirect evidence. In fact, in the earlier study by Kraml *et al*. [18], iron was usually administered in combination with isolated LDL. Another early study by De Kimpe *et al*. [19] showed that in differentiated THP‐1 cells, reactive oxygen species selectively upregulate one splicing isoform of SR‐AI. The synergy between body iron and LDL levels is also supported by epidemiological evidence [20].

In the course of atherogenesis, LDL is generally assumed to be oxidized in the blood vessel wall outside the cells. However, this assumption is questionable, at least according to a series of studies by Leake *et al*. (e.g., [21, 22]), which suggested that LDL is more likely oxidized inside cells by an iron‐dependent process within the lysosomes of macrophages.

This study aimed to test our working hypothesis that if THP‐1 monocytes receive more iron in combination with LDL, enhanced intralysosomal LDL oxidation will lead to the induced expression of SR‐A and induce the cells into a more proatherogenic state.

An abstract of the presented data has been already published [23].

## Materials and methods

### Materials

The low‐/very low‐density lipoprotein (LDL/VLDL) Purification Kit was manufactured by Cell Biolabs, Inc. (San Diego, CA, USA). The Zeba™ Desalt Spin Columns and M‐PER Mammalian Protein Extraction Reagent were from Pierce™ (brand of Thermo Fisher Scientific, Waltham, MA, USA). Iscove's modified Dulbecco's medium (IMDM) with and without the acid–base indicator phenol red was obtained from Gibco™ (also a brand of Thermo Fisher Scientific). Fetal bovine serum for cell culture (FBS, South America origin, endotoxin < 0.7 EU·mL^−1^) was provided by PAN Biotech GmbH (Aidenbach, Germany). The RNAprotect Tissue Reagent, RNeasy Plus Mini Kit, QuantiTect Reverse Transcription Kit, HiPerFect Transfection Reagent, silencing RNAs (siRNAs) against NCOA4, and nonsilencing control siRNA were all purchased from Qiagen (Hilden, Germany). The master mix used for quantitative PCR was the Power SYBR™ Green PCR Master Mix from Thermo Fisher Scientific.

The polyvinylidene difluoride (PVDF) western blotting membrane was Immobilon‐P from Millipore (part of Merck KGaA, Darmstadt, Germany). The anti‐macrophage scavenger receptor 1 (MSR1, D8K4E) rabbit monoclonal antibody (#17275), the anti‐glyceraldehyde 3‐phosphate dehydrogenase (GAPDH, 14C10) rabbit monoclonal, horse radish peroxidase (HRP)‐conjugated antibody (#3683), and the anti‐rabbit IgG HRP‐linked antibody (#7074) were purchased from Cell Signaling Technology, Inc. (Danvers, MA, USA). Sodium dodecyl sulfate (SDS) was obtained from SERVA Electrophoresis GmbH (Heidelberg, Germany). The WesternBright™ Quantum chemiluminescent HRP substrate was obtained from Advansta Inc. (San Jose, CA, USA).

The lipid peroxidation‐sensitive fluorescent probe BODIPY™ 581/591 C11 was manufactured by Invitrogen™ (brand of Thermo Fisher Scientific). Bafilomycin, butylated hydroxytoluene (BHT), chlorotrimethylsilane, cholesterol‐d7, deferoxamine (DFO), 1,1′‐Dioctadecyl‐3,3,3′,3′‐tetramethylindocarbocyanine perchlorate (DiI), n‐hexane (LiChrosolv), human serum, human holotransferrin, N,O‐bis(trimethylsilyl)acetamide, phorbol 12‐myristate 13‐acetate (PMA), the Pur‐A‐Lyzer Midi Dialysis Kit, pyridine, 2‐[(3‐aminopropyl)amino]ethanethiol dihydrochloride (WR‐1065), and all other common chemicals and reagents were purchased from Merck KGaA (Darmstadt, Germany).

### LDL isolation and oxidation

A precipitation‐based LDL/VLDL Purification Kit (Cell Biolabs, Inc.) was used for rapid isolation of fresh LDL before each experiment. Aliquots of commercially available pooled normal, lipid‐rich human serum were stored at −20 °C. At the beginning of isolation, an aliquot of human serum was thawed and centrifuged (twice at 10 000 ***g*** for 10 min at 4 °C) to separate chylomicrons; LDL was then isolated from 10 mL of the infranatant according to the manufacturer's instructions. The obtained suspension of LDL was extensively dialyzed (20 h) against 0.4 L of phosphate‐buffered saline (PBS) containing 1 mm EDTA (PBS‐EDTA) at 4 °C. The next day, the protein content was determined by a modified Lowry's method [24], and the concentration was adjusted to 5 mg protein·mL^−1^ with PBS‐EDTA. The cholesterol content was measured by Chol 250 BIOLATEST, Erba Lachema (Brno, Czech Republic). The suspension of LDL was sterilized by membrane filtration with a 0.2 μm PVDF filter prior to use.

For oxidation, the isolated LDL (5 mg of protein·mL^−1^) was transferred to PBS without EDTA by Zeba Spin Desalting Columns and incubated with 40 μm copper sulfate for 4 h at ambient temperature. The presence of LDL oxidation was confirmed spectrophotometrically by measuring conjugated dienes as an increase in absorbance at 234 nm [2]. The absorbance increased from 0.6115 ± 0.0873 for native LDL to 0.9509 ± 0.1148 for oxidized LDL (measured at 50 μg·mL^−1^, *N* = 7).

### Cell culture

The human monocytic cell line THP‐1 [25, 26] was purchased from American Type Culture Collection (ATCC, Manassas, VA, USA). The cells were cryopreserved in liquid nitrogen and propagated as needed. The cumulative age *in vitro* was monitored, and care was taken to keep it within approximately 110 days [27]. The THP‐1 cells were grown as a suspension culture in IMDM supplemented with 10% (v/v) FBS in flat‐sided untreated (hydrophobic) tissue culture flasks under controlled physiological conditions (in an atmosphere of 95% humidified air and 5% CO_2_ at 37 °C). The batch of FBS was chosen with special consideration of the minimal endotoxin concentration. Every 2–3 days, the cells were counted, viability was checked by Trypan Blue staining [28], and the cells were centrifuged (130 ***g***, 7 min, 4 °C) and replated in new medium at a density of 2 × 10^5^ cells·mL^−1^. The cell viability measured by Trypan Blue exclusion was approximately 98%. The mean population doubling time [28] was 36.8 h. No marked long‐term shifts in these parameters could be observed throughout the whole project.

In the initial experiments, 2 × 10^6^ THP‐1 cells in 2 mL of IMDM without phenol red (IMDM‐PR−) were dispensed into small untreated cell culture Petri dishes. Phenol red was avoided because it is a potential inhibitor of LDL oxidation [29]. The cells were treated with and without freshly isolated native LDL (250 μg of protein·mL^−1^), oxidized LDL (250 μg of protein·mL^−1^), ferric ammonium citrate (8 μm Fe), or 4 μm holotransferrin (8 μm Fe, Fe/protein = 2) for up to 72 h. All the solutions were sterilized by filtration through a 0.22 μm PVDF filter prior to use. After incubation, the cellular suspensions were transferred to Falcon tubes on ice, allowed to cool for 15 min, and centrifuged (130 ***g***, 7 min, 4 °C). A 500 μL portion of each supernatant was combined with 100 μL of 0.6% (w/v) bovine serum albumin in PBS and kept at −80 °C until determination of cytotoxicity could be performed. The remaining supernatants were removed, and the cell pellets were resuspended in RNAprotect Tissue Reagent. The samples were stored at −20 °C until RNA isolation.

In later experiments, THP‐1 cells (2 × 10^6^ cells in 2 mL of IMDM‐PR−) were incubated in small untreated Petri dishes with and without freshly isolated native LDL (250 μg of protein·mL^−1^) for 24 h, followed by centrifugation, resuspension of the cells in fresh prewarmed IMDM‐PR−, and treatment with WR‐1065 (10 μm), BHT (20 μm), bafilomycin (100 nm), and/or oxidized LDL (20 μg of protein·mL^−1^). After another incubation for up to 24 h, the cells were processed in various ways (detailed below) for the determination of cytotoxicity and RNA isolation, western immunoblotting of SR‐A, chromatography of lipids, and assessment of lipoperoxidation by fluorescent probes.

### Cytotoxicity assay

The cellular toxicity of our treatments was assessed as the leakage of lactate dehydrogenase (LDH) into the culture medium with a kinetic assay based on Warburg's optic test [30]. As described above, samples of cellular supernatants were supplemented with 0.1% bovine serum albumin (w/v) and stored at −80 °C. Before use, the samples were slowly thawed, vortexed, and kept on ice. For the LDH measurement, 3 mL of 1.2 mm sodium pyruvate in 50 mm K‐phosphate buffer pH 7.5, prewarmed to 37 °C, was measured in a quartz cuvette, and then 50 μL of 13 mm NADH (for a final concentration of 0.2 mm) and 100 μL of the sample were added, followed by mixing. The absorbance at 340 nm was recorded every 30 s for 10 min at 37 °C via a Hewlett Packard Diode Array Spectrophotometer 8452A (Hewlett‐Packard Co., Palo Alto, CA, USA) equipped with a tempered cell holder. The slope (ΔA340 nm·s^−1^) was obtained by linear regression of zero‐order data and converted to the catalytic activity of LDH (μkat·L^−1^) with an extinction coefficient for NADH of ε = 6.22 × 10^3^ m ^−1^·cm^−1^.

### Quantitative PCR

Total cellular RNA was isolated with an RNeasy Plus Mini Kit. Then, 1 μg of total RNA was reverse transcribed to cDNA with a QuantiTect Reverse Transcription Kit. The primers used for quantitative PCR (qPCR) were designed with primer‐blast software, which is available online and obtained from Generi Biotech s.r.o. (Hradec Králové, Czech Republic). With each new primer pair, the optimal concentrations for PCR were determined, and the size of the PCR product was verified by agarose gel electrophoresis. The efficiency of qPCR was also determined with a range of template amounts. The following primers were used:

TATA box binding protein (TBP):

F 5′‐GGGTTTCTGGTTTGCCAAGA‐3′; R 5′‐CCAGTGCCATAAGGCATCATT‐3′.

Macrophage scavenger receptor A, transcript variant I (SR‐AI):

F 5′‐CAGGTCGTCTGTAGGAGCTT‐3′; R 5′‐ACACTTCATTCAGCCATATTGGA‐3′.

Macrophage scavenger receptor A, transcript variant II (SR‐AII):

F 5′‐GACCAAAAGGCCAGAAAGGG‐3′; R 5′‐GCCCAACCCACCTGATCTTA‐3′.

Nuclear receptor coactivator 4 (NCOA4):

F 5′‐ATTACTCTGCGCCAGACCAT‐3′; R 5′‐GGGACAGCTACAATACCGGA‐3′.

For all the qPCR analyses, the ViiA7 Real‐Time PCR System (Applied Biosystems™, currently a brand of Fisher Scientific) was used. The amplification reactions were performed in duplicate or triplicate in 96‐well plates. The mixtures (20 μL total volume) contained Power SYBR™ Green PCR Master Mix, 300 nm of each specific primer (only reverse primers for SR‐AII were used at 900 nm), and 50 ng of the cDNA template. The TBP gene was included in each plate as a reference gene. After initial denaturation at 95 °C for 10 min, the program for qPCR amplification continued with 40 cycles, each consisting of 15 s at 95 °C and 1 min at 60 °C, and finished with melting curve analysis, which was useful as a check of specific amplification. Only the Ct values of samples with a single specific PCR product were considered. To evaluate the obtained Ct values, efficiency correction was applied, technical replicates were averaged, and the data were normalized to the expression of the reference gene (TBP), scaled to the mean of the controls, and finally expressed as log2 values.

### Western immunoblotting of SR‐A

The cells (2 × 10^6^ cells per 2 mL IMDM‐PR− in untreated Petri dishes) were incubated with and without native LDL (250 μg of protein·mL^−1^) for 24 h, resuspended in fresh IMDM‐PR− and treated with and without 10 μm WR‐1065 for another 24 h. To prepare samples for western blotting, the cellular suspensions were transferred from Petri dishes to Falcon tubes on ice and allowed to cool. Then, 5 mL of ice‐cold PBS‐EDTA was added to each sample, and the suspensions were mixed by turning them upside down, followed by centrifugation (130 ***g***, 7 min, 4 °C). The supernatants were removed, and the cell pellets were resuspended in 10 mL of ice‐cold PBS‐EDTA and centrifuged again. The cell pellets were lysed in 400 μL of M‐PER buffer supplemented with protease inhibitors and 0.5 mm EDTA, as directed by the manufacturer. An aliquot of each extract was subjected to denaturation with 1% (w/v) SDS and 0.125 m dithiothreitol at 100 °C for 10 min and stored frozen until use. The protein concentration of another portion of each extract was measured by the Bradford assay.

For western immunoblotting of SR‐A, denatured samples (10 μg of protein) were applied to a discontinuous 4.5%/10% SDS‐polyacrylamide gel. The proteins were resolved via electrophoresis (15 mA per gel, approximately 2 h at room temperature) and blotted by a semidry transfer (65 mA per gel, 30 min) onto a PVDF membrane. The blots were blocked for 1 h with 5% (w/v) skim milk in Tris‐buffered saline with 0.05% (w/v) Tween 20 (TBST) and incubated with an antibody against SR‐A diluted 1 : 1000 in 1% (w/v) skim milk in TBST overnight at 4 °C. The next day, the blots were washed with TBST and incubated with an HRP‐conjugated anti‐rabbit antibody diluted 1 : 2000 in 1% (w/v) skim milk in TBST for 1 h at room temperature. The blots were washed with TBST again and developed with the WesternBright™ Quantum chemiluminescent HRP substrate, as directed by the manufacturer. The chemiluminescence signal was read with a Fusion FX7 (Vilber Lourmat, Marne‐la‐Vallée, France) instrument. The blots were then developed again with an HRP‐conjugated antibody against GAPDH (diluted 1 : 5000) as a loading control.

For the quantification, each series of samples was run on the gel twice, and two gels with the same samples were run in parallel, resulting in four technical replicates. The intensities of the SR‐A and GAPDH chemiluminescent signals were evaluated with vilber lourmat bio‐1d software version 15.03. The data from the technical replicates were averaged, and the GAPDH signal was normalized to the mean value of each series of samples. The results are expressed as the SR‐A/GAPDH ratio and were normalized to the mean value obtained for the control samples.

### Adhesion assay

THP‐1 cells were seeded in ordinary (treated, hydrophilic) 6‐well tissue culture plates at a density of 2 × 10^6^ cells per 2 mL of IMDM‐PR− in each well. Two wells from the plate served as controls, two others received freshly isolated LDL (250 μg of protein·mL^−1^), and in the last two wells, the cellular differentiation and adhesion [26] were induced by the addition of 0.25 μm PMA. The plates were incubated for 48 h. Cellular adhesion was examined by counting the adherent cells by microscopy and (in other plates) by determining the total protein concentration. For microscopy, the nonadherent cells in suspension were discarded, and the wells with adhering cells were quickly washed twice with 2 mL of prewarmed IMDM‐PR−. Four random photographs at 200× magnification, each from one quadrant, were taken from each well with an inverted Leica DM IL HC microscope (Leica Microsystems GmbH, Wetzlar, Germany) equipped with a camera and phase contrast. For quantification, the cells in the photos were counted, and the counts were averaged and expressed as a percentage of the number of adherent cells in the wells treated with PMA, which served as positive controls with maximal adhesion.

Alternatively, the number of adherent and nonadherent cells in the 6‐well plates was estimated by measuring the total protein concentration. The cellular suspensions were transferred to Falcon tubes on ice, and the wells were quickly washed with 5 mL of ice‐cold PBS‐EDTA per well. Care was taken to avoid drying the wells, and this wash was saved and added to the suspensions in the Falcon tubes. Another 5 mL of ice‐cold PBS‐EDTA was added to the contents of the Falcon tubes. Finally, the adherent cells in the wells were scraped into 500 μL of M‐PER containing protease inhibitors and 0.5 mm EDTA and lysed as directed by the manufacturer. In the meantime, the cellular suspensions in the Falcon tubes were allowed to cool, the mixtures were centrifuged (130 ***g***, 7 min, 4 °C), the supernatants were removed, and the pellets were lysed in 500 μL of M‐PER solution with inhibitors. Microtubes with cellular extracts were stored frozen at −20 °C until protein measurement could be performed. The total protein concentration was determined by the Bradford assay modified for 96‐well microplates. The data from the technical replicates (wells from one plate) were averaged, and the data were expressed as a percentage of PMA.

### Determination of cellular cholesterol

Following the 48‐h experiment described above (see Cell culture section), the cellular suspensions (containing 2 × 10^6^ cells per 2 mL IMDM‐PR− in untreated Petri dishes) were supplemented with 1 mm BHT (from 100 mm stock in ethanol), transferred to Falcon tubes on ice, and allowed to cool. Ten milliliters of ice‐cold PBS‐EDTA was added to each mixture, the cell suspensions were centrifuged, and the resulting cellular pellets were resuspended in 500 μL of ice‐cold PBS‐EDTA. Fifty microliters of this suspension was taken and kept frozen at −80 °C until analysis could be performed.

Total cellular cholesterol was measured by gas chromatography coupled with mass spectrometry (GC–MS) according to Cohen *et al*. [31]. The sample (approximately 200 000 cells) was resuspended in 3 mL of ethanol, and approximately 2 μg of cholesterol‐d7 as an internal standard was added. Cholesteryl esters were hydrolyzed by adding 390 μL of 8.9 m KOH and incubating for at least 4 h at 37 °C. Then, 2 mL of water and 5 mL of n‐hexane (for liquid chromatography, LiChrosolv) were added, and the mixture was vortexed and centrifuged (2500 ***g***, 5 min, room temperature). The hexane phase was evaporated, and the samples were treated with 40 μL of derivatization mixture (pyridine : N,O‐bis(trimethylsilyl)acetamide : chlorotrimethylsilane, 6 : 2 : 1) for 30 min at 37 °C. One microliter of the mixture was injected (split 1 : 10) into a GC–MS system (Agilent 8890 GC System and Agilent 7000D GC/TQ) equipped with a J&W HP‐5 ms Ultra Inert column (30 m × 0.25 mm, 0.25 μm, Agilent) with helium (2 mL·min^−1^) as the mobile phase. The temperatures were as follows: injection port, 280 °C; column, 200 °C (0–1 min); column, 200–300 °C (1–5 min); and column, 300 °C (5–13 min). The cholesterol concentration was calculated on the basis of the ratios of cholesterol/cholesterol‐d7 derivatives at *m/z* 458.4 and 465.4. In separate aliquots, total protein was determined by the Lowry assay, and the results of cholesterol measurement were expressed as μg of cholesterol per mg of total cellular protein.

### Uptake of DiI‐labeled LDL

For quantification of LDL uptake, we adopted the method described by Teupser *et al*. [32], with modifications. For labeling, 30 mg·mL^−1^ DiI in dimethyl sulfoxide was combined 1 : 100 with 5 mg protein·mL^−1^ of the isolated LDL in PBS‐EDTA (for a final concentration of 60 μg DiI·mg^−1^. prot.), gently mixed, incubated for 20 min at 37 °C, and purified by passing through Zeba Spin Desalting Columns. The obtained LDL‐DiI was sterilized by filtration, and 400 μm BHT (from 100 mm stock in ethanol) was added to a portion of it.

The cells were dispensed into untreated Petri dishes as usual (2 × 10^6^ cells per 2 mL IMDM‐PR−) and incubated for 24 h with and without DiI‐LDL or DiI‐LDL‐BHT (250 μg of protein·mL^−1^). Following incubation, the cell suspensions were centrifuged, resuspended in fresh IMDM‐PR−, and incubated for an additional 24 h. The cells were subsequently transferred to Falcon tubes on ice. After cooling, 10 mL of ice‐cold PBS‐EDTA was added to each tube, the suspensions were centrifuged, and the resulting pellets were dissolved in 1 mL of 0.1% (w/v) SDS in 0.1 m NaOH (SDS‐NaOH).

The measurements of DiI fluorescence were carried out in quartz cuvettes at 25 °C using a FluoroMax 3 instrument (HORIBA Jobin‐Yvon S.A.S., France) set to λ_ex_/λ_em_ = 550 nm/565 nm. The samples were diluted with SDS‐NaOH to avoid saturation of the fluorescence signal. In parallel, portions of the unused DiI‐LDL or DiI‐LDL‐BHT were also measured so that the amounts of DiI detected in the cellular samples could be expressed as a percentage of the DiI administered to the cells in LDL.

### Assessment of intracellular lipoperoxidation with fluorescent probe

Following 2 days of incubation with LDL and other substances as described above (Cell culture section), the cellular suspensions were centrifuged and resuspended in prewarmed IMDM‐PR−. The cells were stained with 5 μm BODIPY C11 in new Petri dishes for an additional 2 h. The samples were subsequently transferred to Falcon tubes, cooled on ice, centrifuged (130 ***g***, 7 min, 4 °C), and resuspended in double the volume of IMDM‐PR−. Each cellular suspension was transferred to a quartz cuvette with a magnetic stirrer, and the fluorescence spectrum was recorded at 25 °C with a FluoroMax‐3 spectrofluorometer set to λ_ex_/λ_em_ = 488 nm/500–600 nm. Lipoperoxidation was assessed from the ratio between the fluorescence intensity of the green channel (520 nm) and the red channel (590 nm). An increase in the green/red ratio indicates probe oxidation and hence increased lipid peroxidation [33].

### TBARS assay

The ability of the antioxidant WR‐1065 to inhibit lipid peroxidation was tested with murine brain homogenate; for this purpose, the classical assay with 2‐thiobarbituric acid (TBA) was used [34]. This test measures the formation of TBA‐reactive substances (TBARS), supposedly malondialdehyde arising from the oxidation of polyunsaturated fatty acids [2]. Frozen mice were purchased from a local pet shop and kept at −80 °C until use. Before the experiment, a mouse was allowed to thaw, and the brain was rapidly excised on ice. A 4% (w/v) brain homogenate in Tris‐buffered saline (TBS, 20 mm Tris/HCl, 0.137 m NaCl, pH 7.6) was prepared with a glass handheld homogenizer. A series of reaction mixtures was prepared on ice, combining 500 μL of brain homogenate and 1000 μL of either TBS buffer, pH 7.6, or 0.15 m acetate buffer, pH 4.5, with further additions of the various antioxidants DFO, BHT, and WR‐1065, all at concentrations of 100 μm, and finally FeCl_2_ at 10 μm. The solutions were mixed and incubated at 37 °C for 30 min. The reactions were terminated by adding 500 μL of 4.6 m HClO_4_, the mixtures were clarified by centrifugation (3000 ***g***, 10 min, 4 °C), and 1 mL portions of the supernatants were transferred to new test tubes on ice. In addition, two 1,1,3,3‐tetramethoxypropane (TMP) standard solutions of 9.06 and 3.02 μm were prepared. Finally, 100 μL of 0.8% TBA, prepared according to De Aguilar Diaz Leon and Borges [35], was added to each solution, and the tubes, which were closed with glass spheres to minimize evaporation, were heated in a boiling water bath for 30 min. Once cooled, the absorbance at 532 nm was measured. The absorbances of the TMP standards, which quantitatively yield malondialdehyde upon heating, were used to convert the sample absorbances to μm malondialdehyde/TBARS.

### NCOA4 knockdown

For the experiment with siRNAs against NCOA4, we used the HiPerFect Transfection Reagent provided by Qiagen and adopted the manufacturer's protocol for suspension cell cultures. Briefly, 24 h before transfection, the cells were replated at a density of 3 × 10^5^ cells·mL^−1^ in IMDM containing 10% FBS to ensure the exponential phase of culture growth. Two different lyophilized siRNAs targeted at human NCOA4 and the control nonsilencing siRNA were dissolved in RNase‐free water to a final concentration of 10 μm and were kept frozen between the experiments. The cell viability was checked, the cells were centrifuged and resuspended in IMDM with FBS to a concentration of 2 × 10^6^ cells·mL^−1^, and 100 μL aliquots of this suspension were dispensed into the first two columns of a 24‐well tissue culture plate. Transfection complexes of siRNA and HiPerfect Transfection Reagent were prepared in IMDM without serum and added to the cells according to the manufacturer's instructions. After 6 h of incubation, 400 μL of prewarmed IMDM with FBS was added to each well. The final siRNA concentration was 100 nm. After a total incubation time of 48 h, the cells were collected, centrifuged (130 ***g***, 7 min, room temperature), resuspended in 600 μL of prewarmed IMDM‐PR−, and returned to the corresponding wells. LDL (250 μg of protein·mL^−1^) was added to the wells in the second column, and the plate was incubated for 24 h. To harvest the cells for RNA isolation, the cell suspensions were transferred into cooled Falcon tubes; immediately, RNAprotect Tissue Reagent was added to each well to collect any eventually adherent cells. The cellular suspensions in Falcon tubes were centrifuged, and the pellets were resuspended in RNAprotect as usual and pooled with the RNAprotect in the wells.

### Statistical analysis

The results are presented as means ± SDs. Statistical comparisons were performed by statistical features in the graphical and analytical software originlab 2023b. The statistical methods used are specified in the figure legends.

## Results

### Native LDL‐induced SR‐A expression

**Fig. 1:**
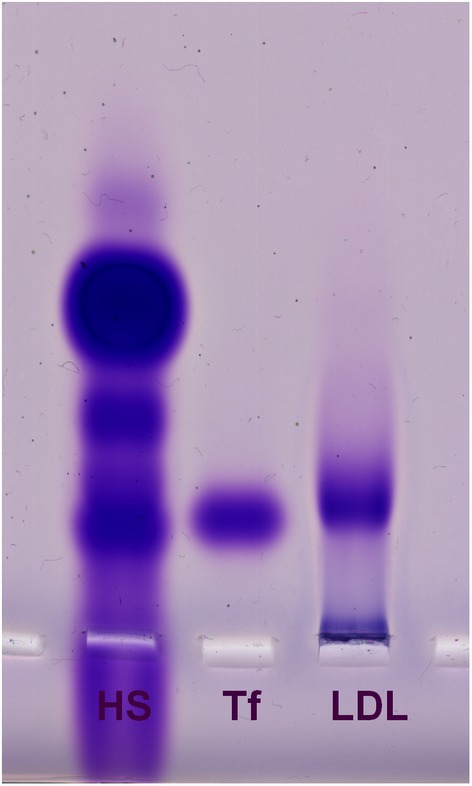
A purity check of isolated LDL. The low‐/very low‐density lipoprotein (LDL/VLDL) fraction was freshly isolated from human serum with a precipitation‐based kit and extensively dialyzed against phosphate‐buffered saline containing 1 mm EDTA. Samples of the original human serum (HS, 45 μg protein), human transferrin (Tf, to indicate the position of β globulins, 2 μg protein), and isolated LDL/VLDL (LDL, 5 μg protein) were loaded onto a 0.5% agarose gel in a 100 mm Tris buffer with 250 mm taurine (pH 8.66). The proteins were resolved by electrophoresis at 90 V for 90 min. The gel was stained for protein with Coomassie Brilliant Blue.

LDL was isolated from human serum with a dextran precipitation‐based LDL/VLDL purification kit, with a typical yield of ~ 4.5 mg protein per 10 mL of serum. The cholesterol content was 3.38 ± 0.45 mmol·g^−1^ prot. In some experiments, isolated LDL/VLDL (further referred to as LDL for simplicity) was subjected to electrophoresis in a 0.5% agarose gel to check its purity: a single band, smeared to the front, with the electrophoretic mobility of the β fraction dominated on the electrophoreogram (Fig. 1). A smudge that appeared near the start (Fig. 1) could be residual chylomicrons, remaining despite the effort expended to remove them, or possibly aggregated LDL/VLDL.

**Fig. 2:**
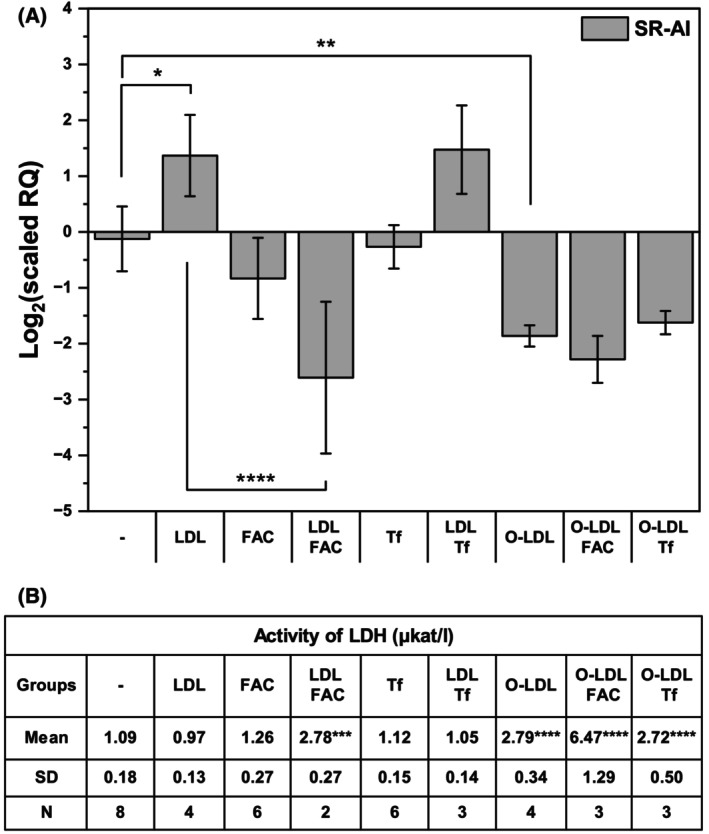
Effects of native LDL, oxidized LDL, ferric ammonium citrate, and transferrin on scavenger receptor AI expression (A) and cell viability (B). THP‐1 monocytes (2 × 10^6^ cells per 2 mL) were incubated with and without native LDL (LDL, 250 μg of protein·mL^−1^), oxidized LDL (O‐LDL, 250 μg of protein·mL^−1^), 8 μm ferric ammonium sulfate (FAC) or 4 μm transferrin (Tf, 8 μm Fe) in culture medium without phenol red for 24 h under physiological conditions. (A) Total RNA was isolated and reverse transcribed, and relative levels of scavenger receptor AI (SR‐AI) were measured by qPCR, with TATA box binding protein (TBP) used as a reference gene. One‐way ANOVA followed by Tukey's *post hoc* test was used to analyze the means ± SDs of three independent experiments (*P < 0.05, **P < 0.01, ****P < 0.0001). (B) Cytotoxicity was determined spectrophotometrically as the release of lactate dehydrogenase (LDH) activity into the culture medium. One‐way ANOVA followed by Sidak's test was used to analyze the means ± SDs of three independent experiments (***P < 0.001, ****P < 0.0001).

**Fig. 3:**
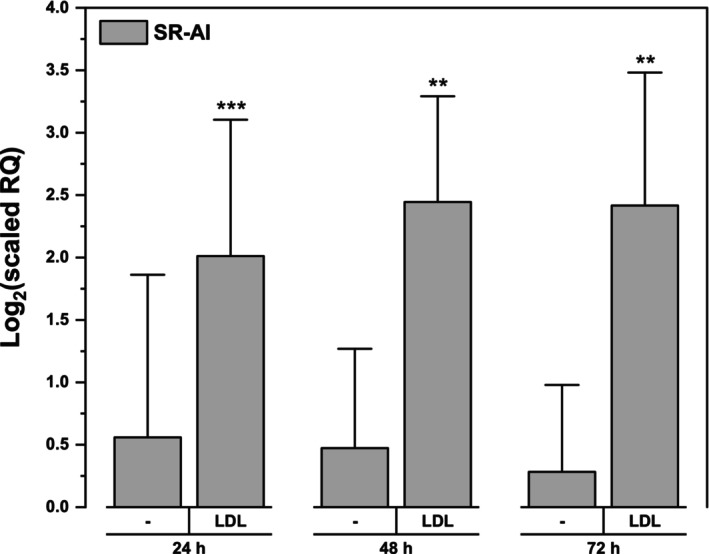
Effect of native LDL on the expression of the scavenger receptor AI. THP‐1 monocytes at a density of 2 × 10^6^ cells per 2 mL were incubated with and without native LDL (250 μg of protein·mL^−1^) in culture medium without phenol red for up to 72 h under physiological conditions. Total RNA was isolated and reverse transcribed to cDNA, and the relative expression of scavenger receptor AI (SR‐AI) was measured by qPCR, with TATA box binding protein (TBP) used as a reference gene. Means ± SDs of four independent experiments (for 24 h of incubation)/three independent experiments (for 48 and 72 h of incubation) were determined. A paired sample *t*‐test was performed (**P < 0.01, ***P < 0.001).

First, we tested our hypothesis that either native or oxidized LDL and iron may lead to synergistic induction of the SR‐AI in THP‐1 monocytes. THP‐1 cells were loaded with iron in the form of transferrin or ferric ammonium citrate, whose cellular uptake mechanisms differ. While transferrin uptake is regulated by the cells themselves, ferric ammonium citrate enters cells in an uncontrolled manner. However, neither form of iron led to increased expression of SR‐AI (Fig. 2A). Contrary to expectations, no increase was detected after loading cells with oxidized LDL (Fig. 2A), likely because of its cytotoxicity (Fig. 2B). Surprisingly, only native LDL alone led to induced SR‐AI expression. This expression was not further increased by iron (Fig. 2A). The SR‐AI expression was roughly fourfold greater than the basal value following 24–72 h incubation with LDL (Fig. 3).

### WR‐1065, a lysosomotropic antioxidant, inhibited LDL‐induced SR‐A expression

**Fig. 4:**
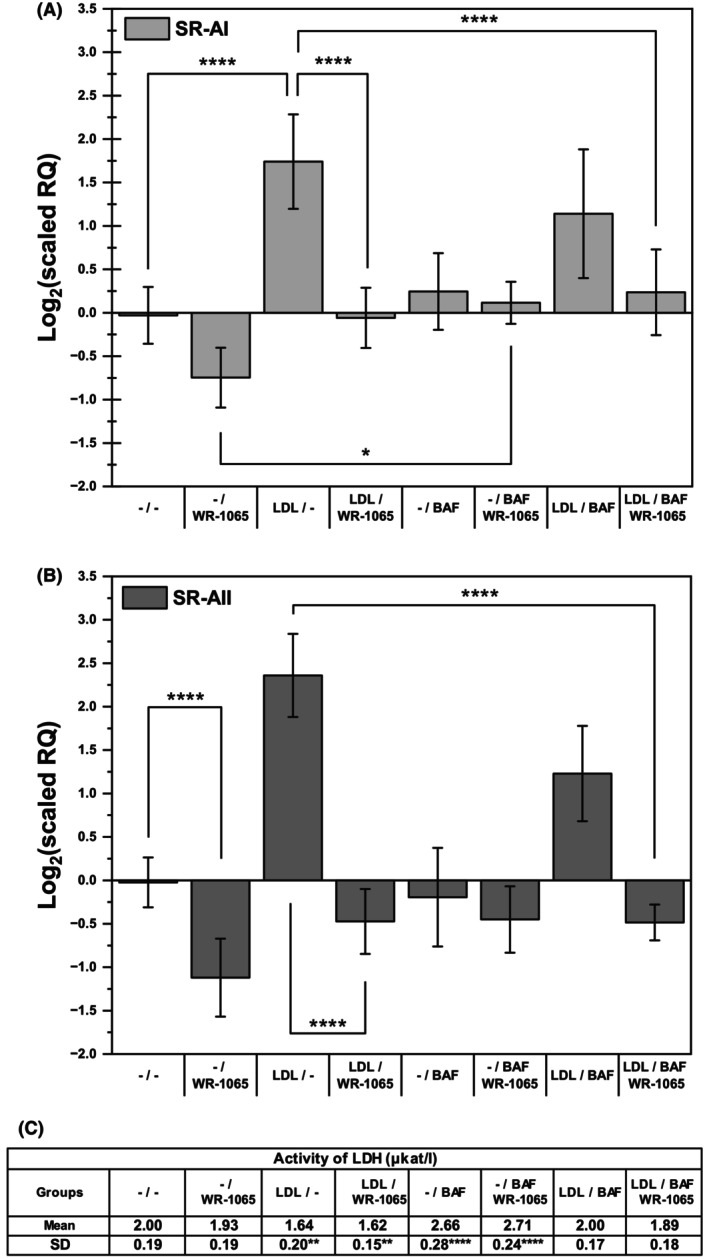
Effects of the antioxidant WR‐1065 and bafilomycin on native LDL‐induced expression of scavenger receptors AI (A) and AII (B) and on cellular viability (C). THP‐1 monocytes (2 × 10^6^ cells per 2 mL) were incubated with and without native LDL (250 μg of protein·mL^−1^) in culture medium without phenol red for 24 h, resuspended in fresh medium and treated with 10 μm lysosomotropic antioxidant WR‐1065 and/or 100 nm bafilomycin (BAF, an inhibitor of the lysosomal proton pump) for an additional day. Total RNA was isolated, reverse transcribed to cDNA, and analyzed by qPCR. The mRNA levels of scavenger receptor AI (A) and AII (B) are expressed relative to those of TATA box binding protein (TBP). One‐way ANOVA followed by Tukey's *post hoc* test was used to analyze the means ± SDs of six independent experiments (*P < 0.05, ****P < 0.0001). (C) The cytotoxicity of the treatments was determined spectrophotometrically as the release of lactate dehydrogenase (LDH) activity into the culture medium. One‐way repeated measures ANOVA followed by Dunnett's test was used to analyze the means ± SDs of six independent experiments (**P < 0.05, ****P < 0.0001).

Because iron loading had no effect, we focused instead on the endogenous cellular iron possibly generated in its redox‐active form inside the lysosomes. This iron can participate in the oxidation of LDL [21] and could also be involved in the subsequent induction of SR‐A expression. The logical approach for targeting cellular iron would be to utilize cell‐permeable iron chelators, but their use was found problematic. Deferoxamine (DFO) induced SR‐A expression (data not shown), apparently as part of the cell differentiation program [36], whereas the more lipophilic and cell‐permeable salicylaldehyde isonicotinoyl hydrazine (SIH) showed high toxicity (not shown), again in agreement with results in the literature [37]. Only WR‐1065, a lysosomotropic antioxidant and a potential iron chelator [38, 39], inhibited both the basal expression of SR‐A and its induction by native LDL (Fig. 4A,B). No cytotoxicity of WR‐1065 was detected (Fig. 4C). We also attempted to reverse the effect of WR‐1065 by altering lysosomal pH with a lysosomal proton pump inhibitor, bafilomycin [40]. The accumulation of WR‐1065 within lysosomes obviously depends on lysosomal acidification. In addition, bafilomycin could also inhibit LDL‐induced SR‐A expression if the catalytic effect of lysosomal iron on lipid oxidation requires an acidic pH [22]. In our experiments, bafilomycin partially reversed the decrease in basal SR‐A expression caused by WR‐1065; its own inhibitory effect on LDL‐induced SR‐A expression did not reach statistical significance (Fig. 4A,B). The response of both known functional splicing variants of SR‐A [7, 19], SR‐AI and SR‐AII, was determined in these experiments, and both followed a similar trend (Fig. 4A,B).

### Native LDL increased SR‐A protein levels and cellular adhesion

**Fig. 5:**
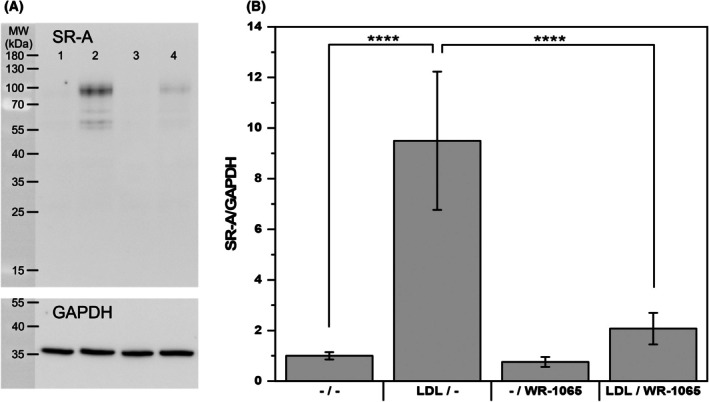
Expression of the scavenger receptor A protein measured by western blotting. (A) A representative western blot is shown. (1) −/−, (2) LDL/−, (3) −/WR‐1065, and (4) LDL/WR‐1065. (B) Densitometric evaluation. THP‐1 monocytes (2 × 10^6^ cells per 2 mL) were incubated with and without native LDL (250 μg of protein·mL^−1^) in culture medium without phenol red for 24 h, resuspended in fresh medium, and treated with and without 10 μm WR‐1065 for another 24 h. The cells were harvested, subjected to SDS (sodium dodecyl sulfate) treatment and SDS‐polyacrylamide gel electrophoresis followed by western blotting and immunodetection of scavenger receptor A (SR‐A) and glyceraldehyde 3‐phosphate dehydrogenase (GAPDH, loading control) as described in the Materials and methods section. The dominant band observed at 70–100 kDa represents SR‐AI, whereas the smaller ~ 60 kDa band most likely represents SR‐AII. Both isoforms were included in the densitometric evaluation. The results are expressed as the SR‐A/GAPDH ratio. One‐way ANOVA followed by Tukey's *post hoc* test was used to analyze the means ± SDs of three independent experiments (****P < 0.0001).

Changes in the amount of synthesized SR‐A were also detected at the protein level by western blotting (Fig. 5A) with densitometric evaluation (Fig. 5B): a ninefold increase in the SR‐A/GAPDH ratio was detected following LDL administration. In line with the qPCR results, WR‐1065 also suppressed the relative amount of LDL‐induced SR‐A almost to the level of the control.

**Fig. 6:**
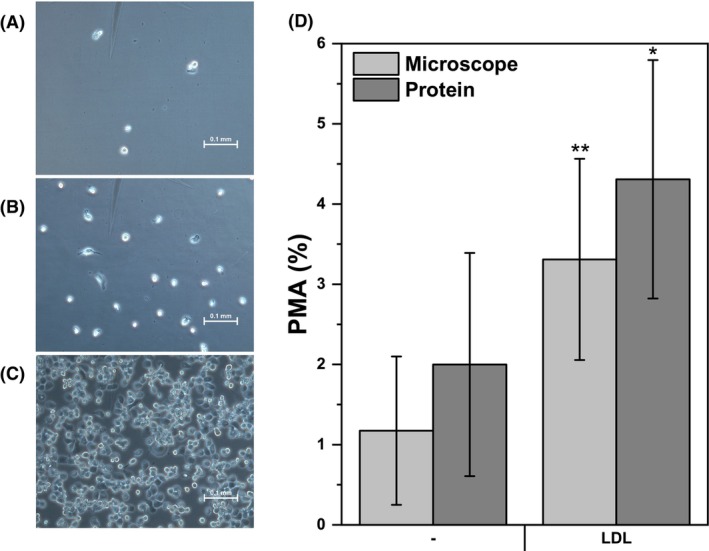
Effects of native LDL on cellular adhesion. Representative microphotographs at a 200× magnification of (A) adherent control THP‐1 cells, (B) cells treated with LDL or (C) cells treated with PMA (phorbol ester that induces cell differentiation—positive control). The scale bars represent 0.1 mm. (D) Graphical representation of the quantitative data obtained from microscopy analysis and total protein determination. THP‐1 cells were incubated in tissue culture 6‐well plates at a density of 2 × 10^6^ cells per 2 mL in each well with and without 0.25 μm PMA and freshly isolated LDL (250 μg of protein·mL^−1^) for 48 h. Nonadhering cells were washed out, four random photos from each well were taken, and the cells were counted. In other plates, total protein from adherent and suspension cells was determined by the Bradford assay. Technical replicates were averaged, and the data from both assays are expressed as percentages of the values after PMA treatment (positive control). A paired sample *t*‐test was used to analyze the means ± SDs of three independent experiments (microscopy)/four independent experiments (total protein concentration) (*P < 0.05, **P < 0.01).

Previous studies have indicated that SR‐A mediates cellular adhesion [41]. The THP‐1 cells grow as a suspension culture in untreated (hydrophobic) culture vessels but display a limited tendency to adhere if they are plated into a usual (treated, hydrophilic) cell culture dish. Therefore, we tested whether cellular adherence to the hydrophilic surface of the cell culture plastic was measurably promoted by native LDL. Indeed, more than three times more adherent cells were present after LDL intake (Fig. 6). The results obtained with a microscope were confirmed by measurement of total protein (Fig. 6D).

### Butylated hydroxytoluene (BHT) inhibited LDL‐induced SR‐A expression

**Fig. 7:**
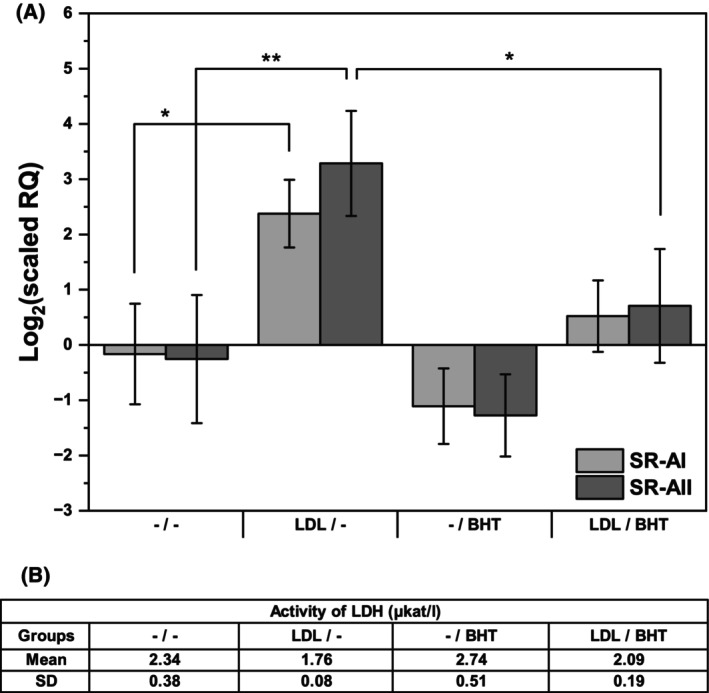
Effects of butylated hydroxytoluene on native LDL‐induced scavenger receptor A expression. THP‐1 monocytes (2 × 10^6^ cells per 2 mL) were incubated with and without freshly isolated native LDL (250 μg of protein·mL^−1^) in culture medium without phenol red for 24 h, resuspended in fresh medium and treated with and without 20 μm butylated hydroxytoluene (BHT) for another 24 h. Total RNA was isolated, and reverse transcription was conducted. (A) Relative mRNA levels of scavenger receptor AI (SR‐AI) and AII (SR‐AII) measured by qPCR and expressed relative to TATA box binding protein (TBP) expression. One‐way ANOVA followed by Tukey's *post hoc* test was used to analyze the means ± SDs of three independent experiments (*P < 0.05, **P < 0.01). (B) Cytotoxicity was determined by measuring the lactate dehydrogenase (LDH) activity released into the culture medium. One‐way repeated measures ANOVA followed by Dunnett's test was used to analyze the means ± SDs of three independent experiments.

Another way to inhibit iron‐dependent lipoperoxidation within cells is the use of the well‐known fat‐soluble, chain‐breaking antioxidant BHT [2]. As shown in Fig. 7A, BHT suppressed the expression of both splicing variants of SR‐A induced by LDL. Although the release of LDH after BHT suggested some cytotoxicity, the differences were not significant (Fig. 7B).

### Native LDL promoted lysosomal lipoperoxidation

**Fig. 8:**
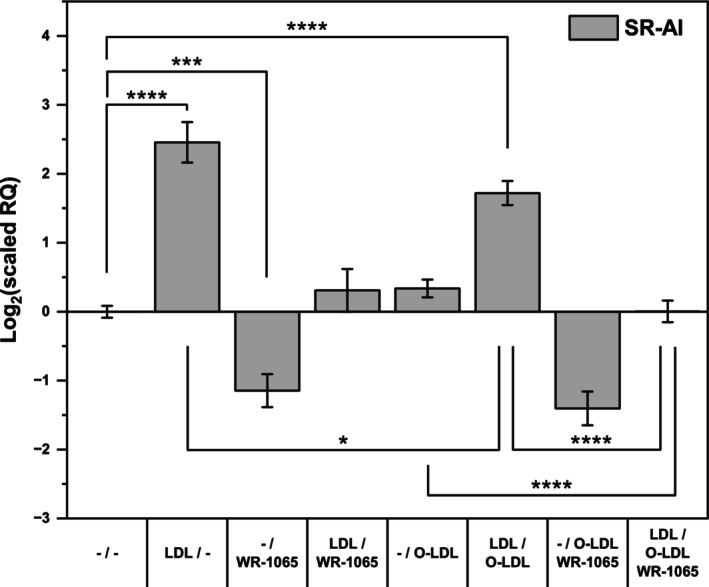
Effects of native LDL, oxidized LDL, and WR‐1065 on scavenger receptor AI expression. THP‐1 cells (2 × 10^6^ cells per 2 mL) were incubated with and without freshly isolated native LDL (250 μg of protein·mL^−1^) in culture medium without phenol red for 24 h. The cells were subsequently resuspended in fresh medium and treated with and without oxidized LDL (O‐LDL, 20 μg of protein·mL^−1^) and 10 μm WR‐1065 for another 24 h. Total RNA was isolated, and reverse transcription was carried out, followed by qPCR. The mRNA levels of scavenger receptor AI (SR‐AI) are expressed relative to those of TATA box binding protein (TBP). One‐way ANOVA followed by Tukey's *post hoc* test was used to analyze the means ± SDs of three independent experiments (*P < 0.05, ***P < 0.001, ****P < 0.0001).

Zhang *et al*. [33] reported that the combination of native LDL with oxidized LDL resulted in considerably more extensive lipoperoxidation in lysosomes. On the basis of this, we hypothesized that mediators of SR‐A induction could already be supplied in oxidized LDL, whereas in the case of native LDL, they must be formed during oxidation in lysosomes. Thus, SR‐A expression should be induced even more if native LDL is administered in combination with a small amount of oxidized LDL. However, as shown in Fig. 8, this combination instead led to a marked decrease in the SR‐AI expression compared with that induced by treatment with native LDL alone. The inhibitory effect of WR‐1065 was unchanged.

**Fig. 9:**
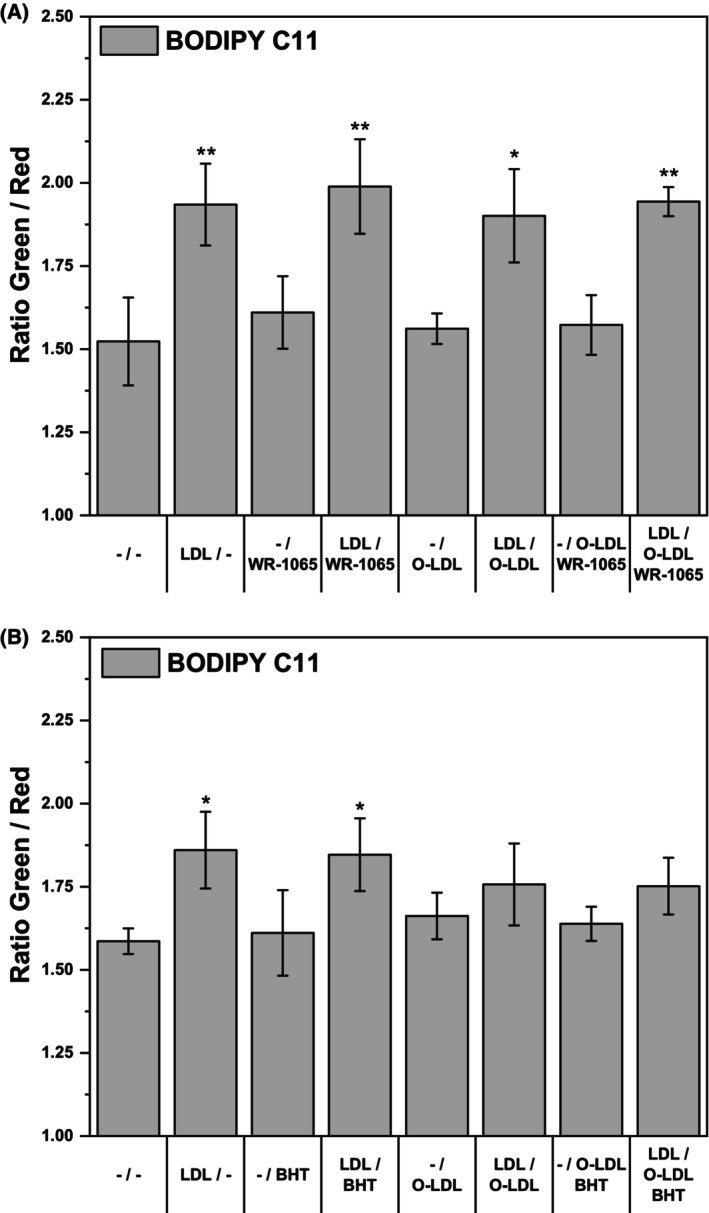
Assessment of lipoperoxidation in THP‐1 monocytes by ratiometric fluorescent probe BODIPY C11. The THP‐1 cells (2 × 10^6^ cells per 2 mL) were incubated with and without freshly isolated LDL (250 μg of protein·mL^−1^) in culture medium without phenol red for 24 h and then resuspended in fresh medium and treated with and without oxidized LDL (O‐LDL, 20 μg of protein·mL^−1^), 10 μm WR‐1065 and 20 μm butylated hydroxytoluene (BHT). After 16 h, the cells were again resuspended in fresh medium and stained with BODIPY C11 (5 μm) for additional 2 h. Lipid peroxidation was assessed by the ratio of the fluorescence intensity in the green channel (520 nm) to that in the red channel (590 nm). Excitation wavelength was 488 nm. One‐way ANOVA followed by Sidak's *post hoc* test was used to analyze the (A) means ± SDs of three independent experiments, (B) means ± SDs of four independent experiments (*P < 0.05, **P < 0.01).

Therefore, we decided to monitor intracellular lipoperoxidation after LDL intake. Its rate was quantified with a lipoperoxidation‐sensitive fluorescent probe, BODIPY C11 (Fig. 9). Indeed, the intracellular peroxidation of lipids was elevated after LDL treatment, but WR‐1065 failed to reduce it, let alone prevent it. Surprisingly, combining native LDL with oxidized LDL did not increase the level of lipoperoxidation detected. Similar experiments were also performed with BHT instead of WR‐1065 (Fig. 9B). Despite being a well‐known lipoperoxidation inhibitor, BHT had no effect on cellular lipoperoxidation, as detected by BODIPY C11.

### Cells demonstrated a very low uptake of native LDL

The same experimental setup shown in Figs 8 and 9 was also used for the quantification of cellular cholesterol in an attempt to assess the uptake of LDL and/or oxidized LDL. The total cellular cholesterol in control cells was 31 ± 4 μg·mg^−1^ protein (*N* = 3), and no statistically significant differences were found for cells treated with LDL and/or oxidized LDL with and without WR‐1065 (data not shown). We then measured the cellular uptake of native LDL fluorescently labeled with DiI, with and without the addition of BHT. Following 24 h of incubation with DiI‐LDL, washing and further incubation for 24 h in medium (corresponding to the condition LDL/− in Figs 7, 8, 9), the amount of DiI found within the cells was only 1.61 ± 0.43% of the original amount if provided in native LDL and 2.43 ± 0.42% if the DiI was contained in LDL supplemented with BHT (data from 3 experiments).

### WR‐1065 really acted on LDL within cells

**Fig. 10:**
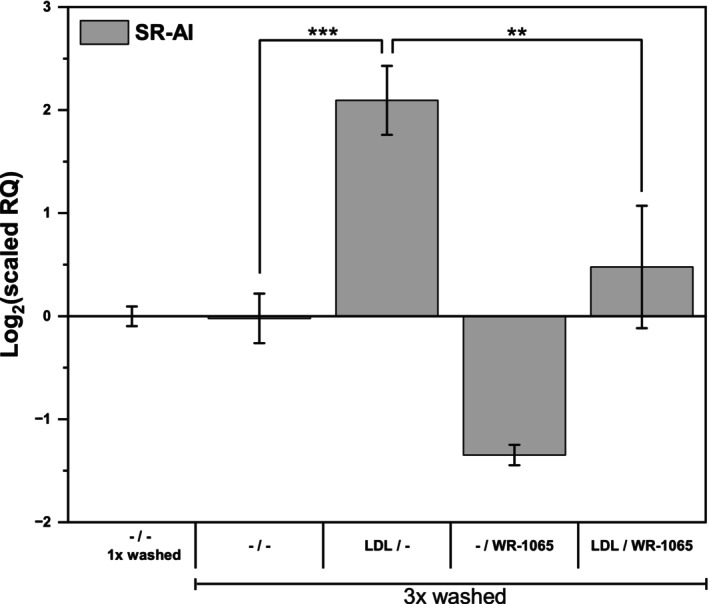
Effects of thorough cell washing on the LDL‐induced expression of scavenger receptor AI. THP‐1 monocytes (2 × 10^6^ cells per 2 mL) were incubated with and without freshly isolated native LDL (250 μg of protein·mL^−1^) in culture medium without phenol red for 24 h. After incubation, cells were resuspended in fresh medium as before (the first control) or two times washed in fresh medium with 0.5% (w/v) bovine serum albumin, and then resuspended in fresh medium, that is, three washes in total (all other samples). Subsequently, cells were incubated with and without 10 μm WR‐1065 for another 24 h as indicated. Total RNA was isolated and reverse transcribed to cDNA, followed by qPCR. The mRNA levels of scavenger receptor AI (SR‐AI) are expressed relative to those of TATA box binding protein (TBP). One‐way ANOVA followed by Tukey's *post hoc* test was used to analyze the means ± SDs from three independent experiments (two experiments for the 1× washed control and treatment with WR‐1065 alone, **P < 0.01, ***P < 0.0001).

In the experiments described above, the incubations of cells with LDL and WR‐1065 were separated by resuspending the cells in fresh medium. But what if this separation was incomplete and the antioxidant WR‐1065 in fact acted on some residual LDL left with the cells throughout the procedure? Separate experiments were conducted to address this potential artifact. We noted that isolated LDL formed almost cell‐size aggregates during 24 h of incubation with cells. The aggregated lipoproteins did not sediment by the low‐speed centrifugation used for the cells, but after a single wash some of these aggregates were trapped among the cells. However, when the cells were thoroughly washed three times (with medium containing bovine serum albumin) between the incubations with LDL and WR‐1065, the effects of both treatments on SR‐A expression were very similar to what was observed before with just one wash (Fig. 10). It supports the notion that LDL‐dependent, antioxidant‐inhibitable processes leading to the observed changes in the SR‐A expression actually occur within cells.

### WR‐1065 does not inhibit iron‐stimulated lipoperoxidation

**Fig. 11:**
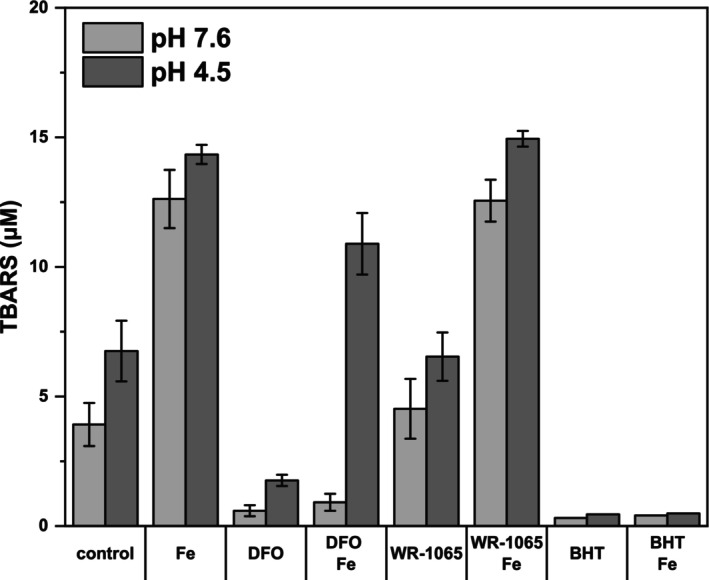
Effects of selected antioxidants on iron‐stimulated lipoperoxidation. Murine brain homogenate either in Tris‐buffered saline, pH 7.6, or in 0.1 m acetate buffer, pH 4.5, was incubated at 37 °C for 30 min in the presence of the antioxidants deferoxamine (DFO), butylated hydroxytoluene (BHT) and WR‐1065, all at 100 μm, and 10 μm FeCl_2_, as indicated. Lipid peroxidation was assessed as the production of thiobarbituric acid‐reactive substances (TBARS). Data combined from three independent experiments are shown (the acetate buffer pH 4.5 was employed in two experiments, and the effect of BHT was tested only once).

The efficacy of the antioxidants used as lipid peroxidation inhibitors was tested through a classic TBARS assay with iron‐stimulated lipid peroxidation of murine brain homogenate (Fig. 11). The reaction took place at an approximately physiological pH of 7.6 and a lysosomal pH of 4.5. BHT effectively prevented the formation of TBARS under both conditions. DFO inhibited lipoperoxidation only at the neutral pH. However, WR‐1065 failed to protect lipids from peroxidation at either pH. On the basis of these results, we dispute the ability of WR‐1065 to act as an inhibitor of lipid peroxidation and question its potency to sequester iron and limit its redox activity.

### LDL‐induced SR‐A expression was dependent on lysosomal iron

**Fig. 12:**
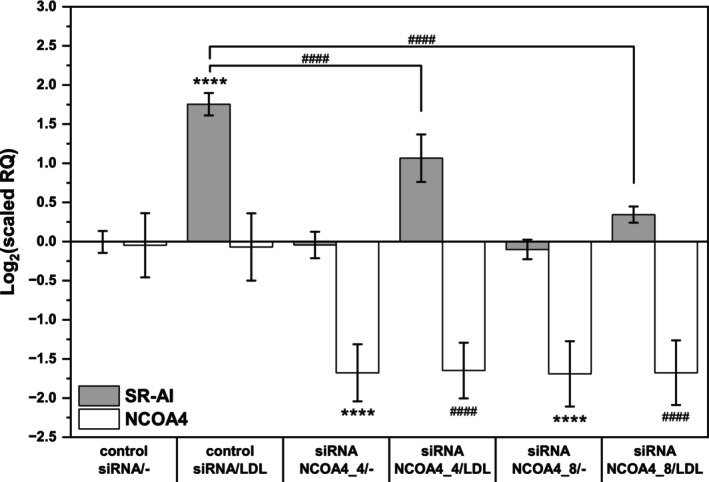
Effect of NCOA4 knockdown on the LDL‐induced expression of the scavenger receptor AI. THP‐1 cells in the exponential phase of growth (2 × 10^5^ cells per well) in the usual culture medium with phenol red and containing 10% fetal bovine serum were transfected with 100 nm of two different siRNAs directed against NCOA4 or a nonsilencing control siRNA for 48 h. After transfection, the cells were resuspended in fresh medium without phenol red, and LDL (250 mg of protein·mL^−1^) was added to half of the cellular suspensions. Total RNA was isolated, and reverse transcription was carried out, followed by qPCR. The mRNA levels of scavenger receptor AI (SR‐AI) and nuclear receptor coactivator 4 (NCOA4, the cargo receptor for ferritinophagy) are expressed relative to those of TATA box binding protein (TBP). One‐way ANOVA followed by Tukey's *post hoc* test was used to analyze the means ± SDs of five independent experiments (****P < 0.0001 in comparison with the control siRNA/−; ####*P* < 0.0001 in comparison with the control siRNA/LDL).

Another way to target endogenous iron is to prevent it from entering lysosomes. Growing cells normally maintain adequate lysosomal iron contents by uptake of transferrin, which is present in the fetal calf serum added to the medium. However, under serum‐free conditions during experiments, they are forced to use their iron stores. Cellular iron is universally stored in cytosolic ferritin and can be released and reutilized via a specific type of autophagy known as ‘ferritinophagy’. This process of lysosomal ferritin uptake is mediated by a selective ferritin cargo protein, NCOA4 [42]. The blockade of ferritinophagy by the knockdown of NCOA4 expression with siRNA should, in the absence of serum transferrin, gradually result in iron‐depleted lysosomes. Two different siRNAs directed against NCOA4 were used. As presented in Fig. 12, both siRNAs effectively silenced the NCOA4 expression, and LDL administration did not alter it. Nonsilencing control siRNA did not affect LDL‐induced SR‐A expression. Most importantly, the silencing of NCOA4 in both cases resulted in a significant decrease in LDL‐induced SR‐AI expression. These results provide clear evidence for the involvement of lysosomal iron in the induction of SR‐A expression by native LDL.

## Discussion

Our original hypothesis was that a synergy between iron and intracellular lipids can induce the expression of SR‐A. Therefore, we performed experiments in which THP‐1 cells were treated with both iron as a peroxidation catalyst and isolated LDL as the peroxidation substrate. Iron alone failed to induce SR‐AI expression (Fig. 2), which is in agreement with our previous data (unpublished). Surprisingly, however, native LDL alone was sufficient to induce SR‐AI expression (Figs 2 and 3), with added iron having no effect (Fig. 2). A likely explanation, still within our working hypothesis, is that the cells already had enough iron for SR‐A induction, and the effect could not be further stimulated by increasing the iron supply. Iron is an obvious catalyst of lipid peroxidation in the body [2] and is particularly effective at acidic pH values inside the lysosome [22]. Nevertheless, we sought direct evidence that endogenous iron is involved. The usual strategy would be the usage of cell‐permeable iron chelators, but that approach has proven difficult because of toxicity [37] and/or the promotion of cellular differentiation [36], which in THP‐1 cells includes the induction of SR‐A [26]. Among the substances tested, WR‐1065 [38, 39] appeared to be the best inhibitor of SR‐AI induction by LDL (Fig. 4). However, we found that this substance is not an effective iron chelator, at least at acidic pH in the lysosome (Fig. 11). Ultimately, we attempted to interrupt ferritinophagy with a siRNA against NCOA4, the cargo receptor for ferritin [42]. The lysosomal compartment can obtain iron either from extracellular sources by receptor‐mediated endocytosis of transferrin or from intracellular sources by receptor‐mediated autophagy of ferritin. In the absence of transferrin, the inhibition of ferritinophagy should result in lysosomal iron depletion. This approach was successful (Fig. 12) and provided good evidence that endogenous iron is involved in the induction of SR‐A by native LDL. We can conclude that our working hypothesis is supported by our experimental data. It is possible that if the cells were sufficiently iron‐depleted before the experiment, the addition of iron would work and enhance the SR‐A induction by LDL.

The measurements of total cellular cholesterol, together with the experiments with fluorescently labeled LDL, revealed that the actual cellular uptake of LDL was very small. This finding is not surprising, as the cellular uptake of LDL is known to be tightly regulated. However, even this tiny amount was sufficient to induce SR‐A expression. The possibility of LDL being first oxidatively modified by transition metals in the medium and then taken up by cells via SR‐A or other scavenger receptors needs to be considered. We used medium without transition metals, but some iron could still be released from the cellular debris. Importantly, when the labeled LDL was supplemented with BHT, the uptake was somewhat greater. Together, these findings indicate that the cells took up unmodified LDL via the usual LDL receptors in a regulated manner and not via scavenger receptors.

We tried to aggregate the isolated native LDL by vortexing, which should lead to increased uptake [21, 43] and potentially higher SR‐A expression. We did not directly measure the uptake of vortexed LDL, but no difference in SR‐A expression compared with that of native LDL was found. We noted that our LDL formed aggregates during incubation with cells; it is possible it was prone to aggregation because of the precipitation‐based isolation procedure, regardless of vortexing.

LDL obviously fills the lysosomal compartment, where it can interact with iron and likely becomes oxidized. We have made great efforts to prove the significance of lysosomal peroxidation following LDL treatment. The findings that may serve as evidence for this include (a) inhibition of SR‐A induction in response to LDL by the lysosomotropic antioxidant WR‐1065 (Fig. 4), (b) inhibition by the classical chain‐breaking lipoperoxidation inhibitor BHT (Fig. 7), and (c) measurements with a ratiometric, lipid peroxidation‐sensitive fluorescent probe (Fig. 9). These findings are not without discrepancies, which could all be due to the technical limits of the tools used.

For instance, it is quite difficult to say what WR‐1065 truly does. It is a biologically active derivative of the known radioprotectant amifostine [39]; chemically a thiol with two amino groups that can be protonated and accumulate within lysosomes in a manner that is dependent on their acidification. Theoretically, if lysosomal acidification is inhibited by bafilomycin, an inhibitor of the lysosomal proton pump, it should cancel all effects of WR‐1065 on SR‐A expression. Simultaneously, bafilomycin could inhibit LDL lysosomal processing on its own. According to our results (Fig. 4), bafilomycin canceled the effect of WR‐1065 on basal, but not LDL‐stimulated, expression of SR‐A, and alone did not produce any significant inhibition of the SR‐A expression. It is worth noting that according to literature [40], the used bafilomycin concentration was not sufficient for a complete loss of the proton gradient. It can be calculated [38, 44] that under these conditions, WR‐1065 still accumulates in lysosomes to some extent.

In contrast to the claims in the literature [38, 39], when we tested whether WR‐1065 can act as an inhibitor of iron‐stimulated lipid peroxidation (Fig. 11), we found that it does not. This is not surprising, as thiols are generally not good inhibitors of lipid peroxidation [2], and it can be inferred that WR‐1065 cannot effectively bind iron at acidic pH because of the protonation of both amino groups. Therefore, it is likely simply a lysosomotropic, water‐soluble antioxidant. However, at least the ability of WR‐1065 to accumulate within lysosomes can be presumed, as it is a simple function of the substance structure and dissociation constants, and therefore, the inhibition by WR‐1065 serves as a proof that the critical oxidation events in LDL‐induced SR‐A expression take place within the lysosome. In contrast, BHT was clearly shown to be a lipid peroxidation inhibitor ([2] and Fig. 11), but it is not particularly lysosomotropic. Therefore, the data obtained with WR‐1065 and BHT complement each other.

The results with the fluorescent probe BODIPY C11 are also somewhat difficult to interpret. The probe demonstrated an increase in lipid peroxidation after LDL treatment but failed to show the added presence of oxidized LDL and the effects of antioxidants (Fig. 9), even in the case of BHT, which we can be sure actually acts as a lipid peroxidation inhibitor. However, lipid peroxidation is a multistep process [2], and the partial responsivity of the probe is likely due to its susceptibility to oxidation [45]. If the probe becomes oxidized itself before the lipids of interest, it works as an antioxidant and overestimates lipid peroxidation. Conversely, if the probe is oxidized downstream of the lipid derivatives of interest, it will underreport the extent of damage caused by lipid peroxidation [45]. Moreover, the effects of antioxidants used to study lipoperoxidation will also be altered. In our experiments, the probe could misreport the effects of added antioxidants simply because it was oxidized instead.

In particular, both WR‐1065 and BHT were effective inhibitors of LDL‐induced SR‐A expression even when added after LDL treatment to the cells resuspended in fresh medium. Some of the aggregated lipoproteins can get trapped among the cells during centrifugation, but we can show that the observed effects of LDL and WR‐1065 last even if they are thoroughly separated with three washes of cells (Fig. 10). This finding further proves that the LDL oxidation necessary for SR‐A induction occurs intracellularly. It is possible that the LDL remaining in the medium aggregates due to oxidation by iron liberated from the cells and then loses its ability to activate SR‐A expression, as the LDL oxidized *in vitro* (Figs 2 and 8).

Currently, we do not know what occurs next. We assume generation of some oxidized lipid mediators that move out of lysosomes and induce SR‐A expression. The identity of these mediators merits further research. Interestingly, SR‐A expression was not induced by the LDL pre‐oxidized *in vitro* by copper ions in our experiments, either by a high, toxic dose (Fig. 2) or a low dose (Fig. 8). On the basis of these findings, it can be inferred that the putative lipid mediator is formed within cells but not when LDL is oxidized *in vitro*. Various ways of LDL oxidation are, in fact, quite different and produce a different array of products [46].

The SR‐A receptor exists in two isoforms (both functional) generated by alternative splicing. When we separately assessed SR‐AI and SR‐AII, we found that they both changed in parallel (Fig. 4), indicating that the induction of expression did not occur at (or after) the level of splicing. De Kimpe *et al*. [19] reported different results, likely because they conducted their experiments with differentiated THP‐1 cells.

Importantly, we showed that SR‐A induction also takes place at the protein level (Fig. 5) and has functional consequences; the cells treated with LDL displayed measurably increased adhesion to the cell culture plastic (Fig. 6). The difference between the adhesion of treated and untreated cells might appear minor, but if a chronic condition is researched, even such a subtle effect can be very significant.

## Conclusion

A limited uptake of native but not oxidized LDL by the undifferentiated human monocytic THP‐1 cells results in a markedly induced expression of both functional isoforms of SR‐A. The SR‐A induction is seen both at the level of mRNA and protein and is associated with increased cellular adhesion. As far as it can be researched, the native LDL‐induced SR‐A expression depends on the lysosomal lipid oxidation that is catalyzed by endogenous cellular iron.

This observation represents a potentially important mechanism that can contribute greatly to the proatherogenic effects of iron. If it also occurs in true circulating monocytes, this indicates that if the monocytes continue receiving more iron and LDL, they will express more SR‐A, become more adhesive, and are more easily recruited to the inflamed endothelium. Notably, if SR‐A expression is upregulated by newly endogenously formed oxidized lipids, and their uptake is mediated by the SR‐A receptor, a positive feedback loop is produced within the atherosclerotic lesion, further accelerating its progression. Such a mechanism underscores the importance of lysosomal LDL processing, as advocated by Leake *et al*. [21, 22]. Synergy between LDL levels and iron stores has already been suggested by epidemiological evidence [20], and monocytes of patients with acute coronary syndromes have been found to have increased expression of SR‐A [17]. Further research in this area is warranted.

## Conflict of interest

The authors declare no conflict of interest.

## Peer review

The peer review history for this article is available at https://www.webofscience.com/api/gateway/wos/peer‐review/10.1002/2211‐5463.70048.

## Author contributions

MČ participated in study conceptualization and design, performed the majority of experiments, statistically evaluated the data, prepared figures for publication, and wrote parts of the manuscript. RB did western blotting of SR‐A. ML measured cellular cholesterol by GC–MS. AS contributed to qPCR analyses and cell adhesion assays. JP designed the study, did some experiments, and finalized the manuscript. All authors read and approved the final manuscript.

## Data Availability

The data that support the findings of this study are available from the corresponding author [jan.platenik@lf1.cuni.cz] upon reasonable request.
